# A comprehensive survey of the prevalence and spatial distribution of ticks infesting cattle in different agro-ecological zones of Cameroon

**DOI:** 10.1186/s13071-019-3738-7

**Published:** 2019-10-17

**Authors:** Barberine A. Silatsa, Gustave Simo, Naftaly Githaka, Stephen Mwaura, Rolin M. Kamga, Farikou Oumarou, Christian Keambou, Richard P. Bishop, Appolinaire Djikeng, Jules-Roger Kuiate, Flobert Njiokou, Roger Pelle

**Affiliations:** 1 Department of Biochemistry, Faculty of Sciences, University of Dschang, P.O. Box 67, Dschang, Cameroon; 2 Molecular Parasitology and Entomology Unit, Department of Biochemistry, Faculty of Sciences, University of Dschang, P.O. Box 67, Dschang, Cameroon; 3 Biosciences eastern and central Africa-International Livestock Research Institute (BecA-ILRI) hub, P.O. Box 30709-00100, Nairobi, Kenya; 4 Bioscience, International Livestock Research Institute (ILRI), P.O. Box 30709-00100, Nairobi, Kenya; 5 Special Mission for Eradication of Tsetse Flies, Regional Tsetse Division of Adamawa, B.P 263, Ngaoundere, Cameroon; 6 Veterinary Microbiology and Pathology (VMP), Washington State University, 100 Dairy Road, Pullman, WA 99164 USA; 7 Centre for Tropical Livestock Genetics and Health, The Roslin Institute & Royal (Dick) School of Veterinary Studies, The University of Edinburgh, Edinburgh, Scotland EH25 9RG UK; 8 Laboratory of General Biology, Faculty of Sciences, University of Yaounde I, P.O. Box 812, Yaounde, Cameroon

**Keywords:** Tick-borne diseases, Ticks, Identification, Cattle, *cox*1, Agro-ecological zones

## Abstract

**Background:**

Ticks and tick-borne diseases are a major impediment to livestock production worldwide. Cattle trade and transnational transhumance create risks for the spread of ticks and tick-borne diseases and threaten cattle production in the absence of an effective tick control program. Few studies have been undertaken on cattle ticks in the Central African region; therefore, the need to assess the occurrence and the spatial distribution of tick vectors with the aim of establishing a baseline for monitoring future spread of tick borne-diseases in the region is urgent.

**Results:**

A total of 7091 ixodid ticks were collected during a countrywide cross-sectional field survey and identified using morphological criteria. Of these, 4210 (59.4%) ticks were *Amblyomma variegatum*, 1112 (15.6%) *Rhipicephalus* (*Boophilus*) *microplus*, 708 (10.0%) *Rhipicephalus* (*Boophilus*) *decoloratus*, 28 (0.4%) *Rhipicephalus* (*Boophilus*) *annulatus*, 210 (3.0%) *Hyalomma rufipes*, 768 (10.8%) *Hyalomma truncatum*, and 19 (0.3%) *Rhipicephalus sanguineus.* Three ticks of the genus *Hyalomma* spp. and 33 of the genus *Rhipicephalus* spp. were not identified to the species level. Cytochrome *c* oxidase subunit 1 (*cox*1) gene sequencing supported the data from morphological examination and led to identification of three additional species, namely *Hyalomma dromedarii*, *Rhipicephalus sulcatus* and *Rhipicephalus pusillus*. The finding of the invasive tick species *R. microplus* in such large numbers and the apparent displacement of the indigenous *R. decoloratus* is highly significant since *R. microplus* is a highly efficient vector of *Babesia bovis*.

**Conclusions:**

This study reports the occurrence and current geographical distribution of important tick vectors associated with cattle in Cameroon. It appears that *R. microplus* is now well established and may be displacing native *Rhipicephalus* (*Boophilus*) species, such as *R. decoloratus*. This calls for an urgent response to safeguard the livestock sector in western central Africa.

## Background

Ticks rank first among vectors of diseases affecting livestock globally [1]. Their direct effects on the hosts include anemia and excessive grooming, stress, toxicosis and immunosuppression, which often lead to diminished productivity [2]. Ticks also transmit a great variety of pathogenic microorganisms that cause disease in both humans and livestock [3]. Data on the economic impact of ticks and tick-borne diseases (TBDs) are scarce but it has been estimated that, globally, about US$ 20–30 billion are lost annually due TBDs [4].

In a study conducted in 1982 in Cameroon, approximately 63% of animal mortality in the Wakwa research station situated in the principal cattle rearing region was attributed to TBDs [5]. This situation has seriously constrained attempts to rear high performing exotic dairy cattle breeds which are highly susceptible to tick-borne diseases including babesiosis, ehrlichiosis and dermatophilosis [6].

Increased demand for animal food products in West and Central Africa due to rapid population growth has accelerated transboundary livestock movements for trade across the region. Consequently, there is an increased risk of animal disease transmission [7, 8]. In addition, animal movements in sub-Saharan Africa are also linked to transnational transhumance [9]. This regular movement of herders and their livestock across national boundaries to exploit the seasonal availability of pastures is a socio-cultural phenomenon [10]. It represents the transhumant communities’ key resilience strategy to combat fluctuations and long term change in climate [9]. Unfortunately, disease surveillance at the borders of most sub-Saharan African countries is limited or altogether lacking [11]. This has created a situation that allows the importation of exotic tick species and their pathogens into many countries. The recent finding of the cattle tick *Rhipicephalus* (*Boophilus*) *microplus* in West African countries such as Ivory coast, Burkina Faso, Mali, Togo, Benin and Nigeria is worrisome because this tick species is an efficient vector of *Babesia bovis* which causes a virulent form of babesiosis, the most important tick-transmitted disease of cattle globally [12–14]. It is widely believed that *R. microplus* was introduced into West Africa, seemingly through cattle imported from Brazil and has rapidly spread across the sub-region through transboundary cattle movements [14, 15].

Small-scale livestock keepers play an important role in the livestock industry in developing countries, contributing greatly to food security and rural development [16]. Most small scale livestock farmers cannot afford regular tick control with acaricides, relying on labor intensive manual control of ticks, combined with limited chemical treatment, particularly during the rainy seasons [17].

The few studies undertaken on ticks infesting livestock in Cameroon have been limited to the principal cattle rearing areas, namely the Far North, North, Adamawa and North West regions [5, 17–27]. Areas with low animal densities, including the eastern region bordering Central African Republic (C.A.R.) are understudied, yet this area has been the focus of very extensive livestock movements [28]. Such animal movement can contribute to a shift in the tick ‘population landscape’ [29]. Additionally, it has been demonstrated that the distribution of many species will expand or contract as a consequence to global warming and climate change [30]. Despite the high impact of TBDs on the global economy, there is a lack of reliable data since data on the incidence of TBDs and the distribution map of many tick vectors is either not available for many African countries or are outdated. It is therefore urgent to accurately identify and update the distribution of ticks in order to predict the risk of emergence or re-emergence of TBDs in the sub-region.

Most studies on the identification of ticks are primarily based on morphological characterization. However, this method is challenging because morphological differences between closely related tick species are sometimes difficult to establish, especially when the tick specimens are damaged, engorged or from non-adult instars [31, 32]. Morphological classification also requires entomological expertise which is scarce or non-existent in most of the affected countries in sub-Saharan Africa. Reliance only on morphological identification of ticks can therefore result in misidentification if the entomological personnel are not adequately trained. DNA-based methods including the sequencing of specific loci encoded in the nuclear genome such as the ribosomal internal transcribed spacer (ITS) locus, the mitochondrially-encoded *cox*1 gene and the mitochondrial and nuclear ribosomal small subunit RNA genes (*12S* and *16S*) have been developed as additional tools to support identification based on morphological criteria; this is especially the case for damaged specimens, in which key anatomical features can no longer be discriminated [33–35]. Recent studies on tick identification have demonstrated *cox*1 to be the most reliable and suitable molecular marker for the identification of different ticks at the species level [36].

In the present study, a countrywide cross-sectional survey was undertaken with the goal of assembling baseline data on the distribution of ticks in different agro-ecological zones (AEZs) of Cameroon. The implications for the epidemiology of TBDs and tick control in the country are discussed.

## Methods

### Study area

**Fig. 1:**
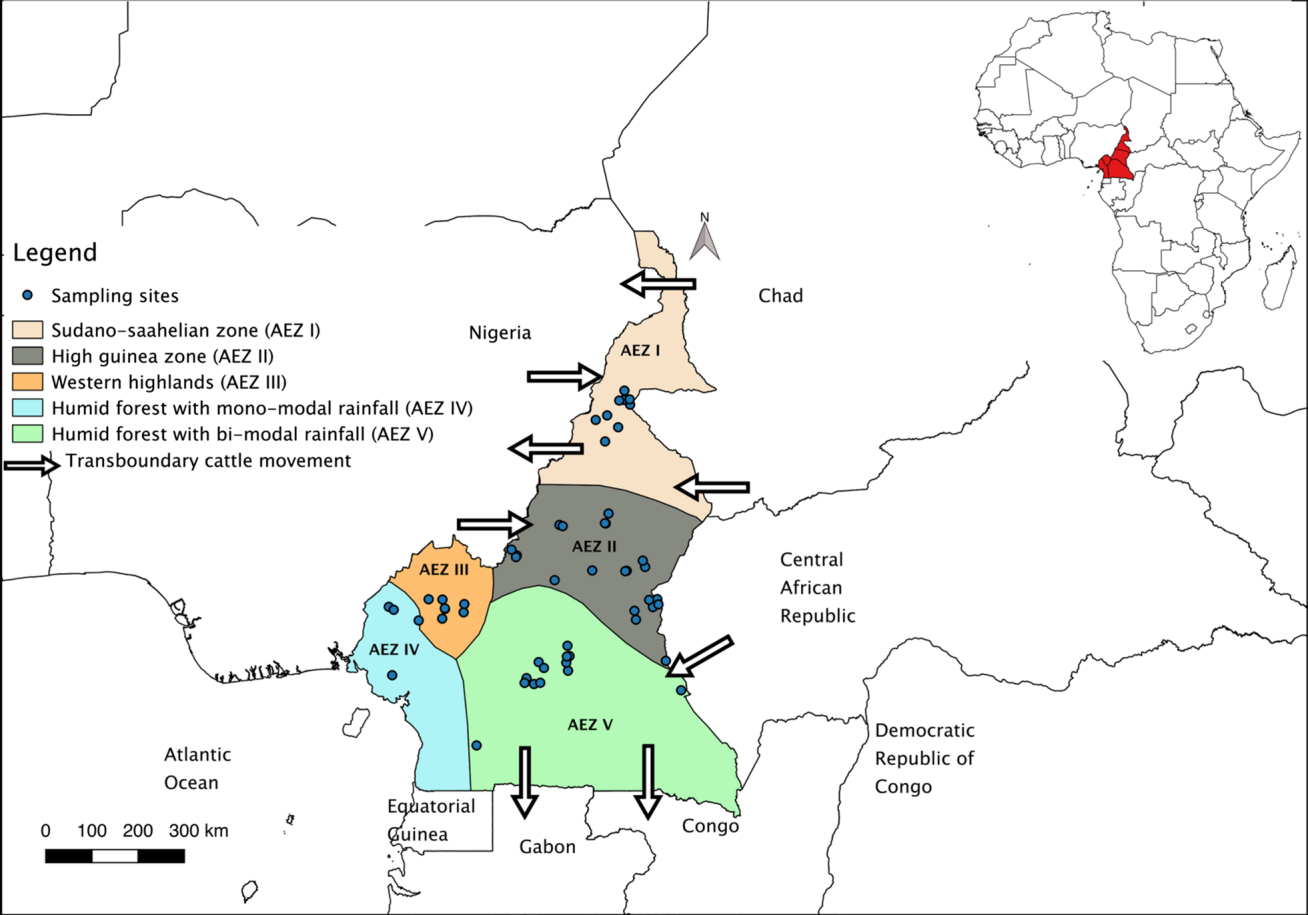
Map showing the five AEZs of Cameroon and the sampling sites

Sampling was conducted at 54 sites across five agro-ecological zones of Cameroon (Fig. 1). The number of sites sampled in each zone was determined by livestock density and willingness of farmers to participate in the survey. Each zone has distinct bioclimatic and environmental characteristics as indicated in Table 1. Briefly, AEZ I (Sudano-Sahelian zone) is characterized by the northern plain with high temperatures, dry savannah and steppes. Accurate statistics on livestock production are not easy to obtain because an appropriate data collection system is lacking [37]. The official cattle population of this zone is estimated at 1.89 million head [38]. AEZ II (High Guinea zone) is dominated by the Adamawa plateau in the center. The cattle population is estimated at 1.13 million head [38]. This region is the main destination of transhumant herders originating from neighboring countries [11]. The vegetation is characterized by savannah and degraded forest. The main physical unit of the AEZ III (Western highlands) is mountain characterized by low temperatures and high rainfall. This area mainly covers the West and the North-west region and hosts approximately 1.98 million head of cattle [38]. AEZ IV (humid forest with mono-modal rainfall) encompasses the coastal lowlands and hosts about 1472 head of cattle [38]. Temperature and annual rainfall are high. AEZ V (humid forest with bi-modal rainfall) is mainly dominated by the southern plateau. This area falls within the tsetse fly zone which constrains cattle rearing. Cattle population is estimated at 276,855 head [38]. Many farmers in this area are refugees originating from high conflict zones in C.A.R. [28].

**Table 1:** Key geographical and climatic characteristics of agro-ecological zones of Cameroon

Agro-ecological zones (AEZs)	Altitude (m)	Annual average temperature (°C)	Annual average precipitation (mm)	Rainy period	Vegetation	Cattle population
Sudano-sahelian zone (AEZ I)	250–500	28.9	923.35	June–August	Dry savannah, steppes	1,898,890
High guinea zone (AEZ II)	500–1500	22.06	1515.3	April–October	Savannah, degraded forest	1,183,137
Western Highlands (AEZ III)	1500–2500	20.64	3080.5	March–October	Savannah, degraded forest	1,989,200
Humid forest zone with mono-modal rainfall (AEZ IV)	0–2500	24	4163.5	March–October	Evergreen forest	1472
Humid forest zone with bimodal rainfall (AEZ V)	400–1000	24.4	2456.8	March–October	Humid forest-savannah mosaic	276,855

Most cattle sampled in the different AEZs were of local breeds (96.67%) with a few exotic (1.83%) or crossbreed (1.5%) animals. Except for few instances where a combination of stall feeding and free grazing is practiced, most cattle are reared under an open grazing system. Small ruminants are found all over Cameroon. They are estimated to comprise 8.2 million animals, with goats outnumbering sheep [37]. Except for AEZ II, where cattle are major ruminant livestock, all the other zones are dominated by sheep and goats [38].

### Sampling and morphological identification of different tick species

Ticks were collected from 601 cattle during a cross-sectional survey conducted from April to August 2016 (during the wet season) in all five agro-ecological zones, covering a total of 54 sites across the country. At each site, an average of 10 to 15 cattle was examined for the presence of ticks. Cattle were restrained and kept standing and all the body parts of the cattle were examined. Only visible adult ticks were collected. Because of their small size, immature stages were not collected. Therefore, none of the collections made from cattle in the present study were intended to be complete. Ticks were plucked using blunt steel forceps. The ticks collected were preserved in 70% ethanol and stored at 4 °C in the lab. The morphological identification of tick species was performed according to the published taxonomic keys using a standard stereomicroscope with a magnification of up to 100× [39]. This identification was conducted in the Tick Unit at the International Livestock Research Institute (ILRI) of Nairobi, Kenya.

For the spatial analysis, the exact site of each tick found attached on the cattle was recorded using a global positioning system data recorder (Garmin eTrex® 20; Garmin, Hampshire, UK). Each location was transferred into QGIS v.2.18 software and plotted on maps.

### Molecular identification of different tick species

To support morphological identification, the identity of ticks was confirmed by molecular analysis of the partial sequence of the *cox*1 gene as described previously [36]. For each species of tick identified morphologically, two to four representative individuals were randomly selected. For specimens identified at the genus level, ticks were grouped, and representatives of each group were selected for molecular analysis (see Additional file 1: Table S1).

### DNA extraction

A total of 30 adult ticks, representative of the species that were identified morphologically, were washed twice in distilled water and air dried for 30 min. Each tick was transferred into a 2 ml sterile micro-tube containing one sterile 4.5 mm glass plating bead (Rattler™ Plating Beads; ZYMO Research, California, USA). The tube was frozen in liquid nitrogen for 5 min and the tick was ground into powder using a Geno-grinder (SPEX Sample Prep; Stanmore, UK). Genomic DNA was extracted using a DNeasy Blood & Tissue Kit (Qiagen; Hilden, Germany) using a protocol recommended by the manufacturer.

### Amplification and sequencing of the *cox*1 gene

A 710 base pair (bp) DNA fragment was generated using the forward primer LCO1490 (5′-GGT CAA CAA ATC ATA AAG ATA TTG G-3′) and the reverse primer HC02198 (5′-TAA ACT TCA GGG TGA CCA AAA AAT CA-3′) as previously described [40]. PCR was performed in a final volume of 50 µl comprising 25 µl of AccuPower® Taq 2x PCR Master Mix (Bioneer; Daejeon, Korea), 50 ng of DNA template and 0.2 μM of each primer. The thermal cycling program consisted of an initial denaturation at 95 °C for 3 min followed by 35 cycles of denaturation at 94 °C for 1 min, annealing at 40 °C for 1 min and extension at 72 °C for 1 min. A final extension was carried out for 10 min at 72 °C. Five microliters of each PCR amplicon were run on a 1.8% agarose gel to check the quality and yield of the PCR product. The amplicons were purified using a QIAquick PCR Purification Kit (Qiagen; Hilden, Germany) following the manufacturer’s protocol. The final concentration of purified PCR product was determined using a spectrophotometer (WPA Lightwave II; Biochrom, Cambridge, UK). The purified amplicons were sequenced at Bioneer using the same forward and reverse primers used to generate the PCR products.

### Sequence editing

Sequences were manually edited and assembled, and consensus sequences were generated and aligned using CLC Main Workbench software v.7.8.1 (CLC bio, Aarhus, Denmark). To confirm the identity of each tick species, the sequences were compared with those available in the GenBank database using the BLASTn program (https://blast.ncbi.nlm.nih.gov/Blast.cgi). For the BLASTn algorithm, a stringent E-value cut-off (10^−6^) was used as described previously [41]. The identity of the query sequence was assigned to the best hit (highest bit score) returned from BLASTn. The query ID was regarded as confirmed when the best hit (highest bit score) had an E-value below 10^−6^.

### Phylogenetic analyses

To determine the relationships between different tick species and infer their evolutionary history, phylogenetic trees were constructed. To build these trees, reference sequences of the *cox*1 gene of ticks were downloaded from the GenBank database. The downloaded sequences were combined with those generated in the present study and the phylogenetic trees were built using a hierarchical likelihood ratio test based on the lowest Bayesian information criterion using MEGA v.7.0. Neighbor-Joining trees were generated and their robustness evaluated using 1000 bootstrap replicates in MEGA v.7.0.

### Statistical analysis

The association between tick burden and environmental factors (agro-ecology) was assessed using generalized linear models (GLM) through the statistical software R v.3.5.3 (https://www.r-project.org). For this, a negative binomial model in which tick burden was the dependent variable and agro-ecological zone was the independent variable was used. The confidence interval (CI) for each AEZ was estimated at 95%.

## Results

### Tick collection and identification

A total of 7091 adult ticks were collected from 601 cattle in 54 sites distributed across the five AEZs (Fig. 1). Ticks were collected from 9, 20, 8, 3 and 14 sites in AEZs I, II, III, IV and V, respectively. On the basis of their morphology, the 7091 ticks were classified into three genera: 4210 (59.4%) *Amblyomma*, 1900 (26.8%) *Rhipicephalus* and 980 (13.8%) *Hyalomma*. These ticks comprised seven species: 4210 (59.4%) *Amblyomma variegatum*, 1112 (15.6%) *R. microplus*, 708 (10.0%) *Rhipicephalus* (*Boophilus*) *decoloratus*, 28 (0.4%) *Rhipicephalus* (*Boophilus*) *annulatus*, 201 (3.0%) *Hyalomma rufipes*, 769 (10.8%) *Hyalomma truncatum* and 19 (0.3%) *Rhipicephalus sanguineus* (see Additional file 2: Figures S1, Additional file 3: Figures S2, Additional file 4: Figures S3, Additional file 5: Figures S4, Additional file 6: Figures S5, Additional file 7: Figures S6, Additional file 8: Figures S7, Additional file 9: Figures S8, Additional file 10: Figures S9, Additional file 11: Figures S10, Additional file 12: Figures S11). Because of morphological similarities among the ticks, 36 specimens were identified to the genus level. This included three ticks of the genus *Hyalomma* spp. and 33 of the genus *Rhipicephalus* spp. The relative abundance and distribution of each tick species for each AEZ is described in Table 2.

**Table 2:** Distribution of tick species per agroecological zone

Tick genus	Tick species	No. of ticks collected (%)	Sex		AEZ I	AEZ II	AEZ III	AEZ IV	AEZ V
					Vegetation type				
					Dry savannah, steppes (%)	Savannah, degraded forest (%)	Savannah, degraded forest (%)	Evergreen forest (%)	Humid forest-savannah mosaic (%)
			Female	Male					
*Amblyomma*	*A. variegatum*	4210 (59.4)	1077	3133	499 (29.9)	2735 (96.5)	243 (43.1)	55 (34.8)	678 (36.3)
*Rhipicephalus*	*R. microplus*	1112 (15.6)	1043	69	0 (0)	5 (0.2)	288 (51.1)	103 (65.2)	716 (38.3)
	*R. decoloratus*	708 (10.0)	686	22	167 (10.0)	73 (2.6)	18 (3.2)	0 (0)	450 (24.1)
	*R. annulatus*	28 (0.4)	13	15	9 (0.5)	0 (0)	2 (0.4)	0 (0)	17 (0.9)
	*R. sanguineus*	19 (0.3)	9	10	5 (0.3)	4 (0.1)	5 (0.9)	0 (0)	5 (0.3)
	*Rhipicephalus* spp.	33 (0.5)	15	18	11 (0.7)	13 (0.5)	8 (1.4)	0 (0)	1 (0.1)
*Hyalomma*	*H. rufipes*	210 (3.0)	59	151	207 (12.4)	3 (0.1)	0 (0)	0 (0)	0 (0)
	*H. truncatum*	768 (10.8)	250	518	766 (46.0)	1 (0)	0 (0)	0 (0)	1 (0.1)
	*Hyalomma* spp.	3 (0)	0	3	3 (0.2)	0 (0)	0 (0)	0 (0)	0 (0)
	Total	7091 (100)	3152	3939	1667	2834	564	158	1868

Analysis of sex ratio was carried out on the five most abundant species, namely *A. variegatum*, *R. microplus*, *R. decoloratus*, *H. rufipes* and *H. truncatum*. The sex ratio of the collected ticks varied with species and skewed towards male, except for *R. microplus* and *R. decoloratus* (Table 3).

**Table 3:** Male: female sex ratio per species

Tick species	Male (♂)	Female (♀)	Total	M:F (♂: ♀)
*A. variegatum*	3133	1077	4210	2.91:1
*R. microplus*	69	1043	1112	0.07:1
*R. decoloratus*	22	686	708	0.03:1
*H. rufipes*	151	59	210	2.56:1
*H. truncatum*	518	250	768	2.07:1

**Fig. 2:**
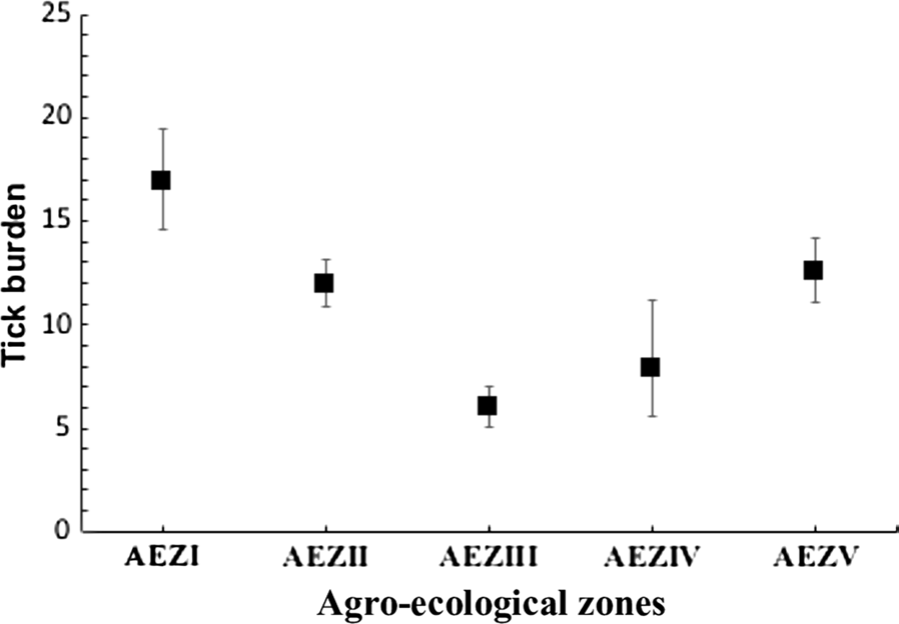
Tick burden on cattle in different agro-ecological zone (AEZ). The mean of each data set is indicated by the black centre square. The bars are confidence intervals at 95%. Values are statistically significant at *P* < 0.0001

The overall mean tick burden per animal (all tick species) fluctuated between 5.9 and 16.8 across the AEZs. Mean tick burden per infested animal was significantly high in AEZ I (16.83, 95% CI: 14.53–19.50) and AEZ V (12.58, 95% CI: 11.10–14.16) compared to other AEZs (*P* < 0.0001) (Fig. 2).

To confirm results from the morphological identification, the partial *cox*1 gene was sequenced and analyzed. Specimens from each tick species were randomly selected for molecular analysis. Specimens of *Hyalomma* spp. and *Rhipicephalus* spp. ticks were grouped by similarity and representative individuals from each group were randomly selected for molecular analysis. A total of 30 specimens were sequenced and the BLASTn analysis was performed. The results from molecular identification are summarized in Additional file 1: Table S1. BLASTn returned best hits that matched the morphological results with identity varying between 87 and 100%. Molecular analysis revealed that among the three specimens of the genus *Hyalomma* spp., two were *Hyalomma dromedarii* and one was *H. truncatum*. For *Rhipicephalus* spp. seven specimens were analyzed among which two were identified as *Rhipicephalus pusillus*, one as *Rhipicephalus sulcatus* and four as *Rhipicephalus sanguineus*. Sequences were deposited in NCBI GenBank (accession numbers MK648401-MK648422, see Additional file 1: Table S1).

**Fig. 3:**
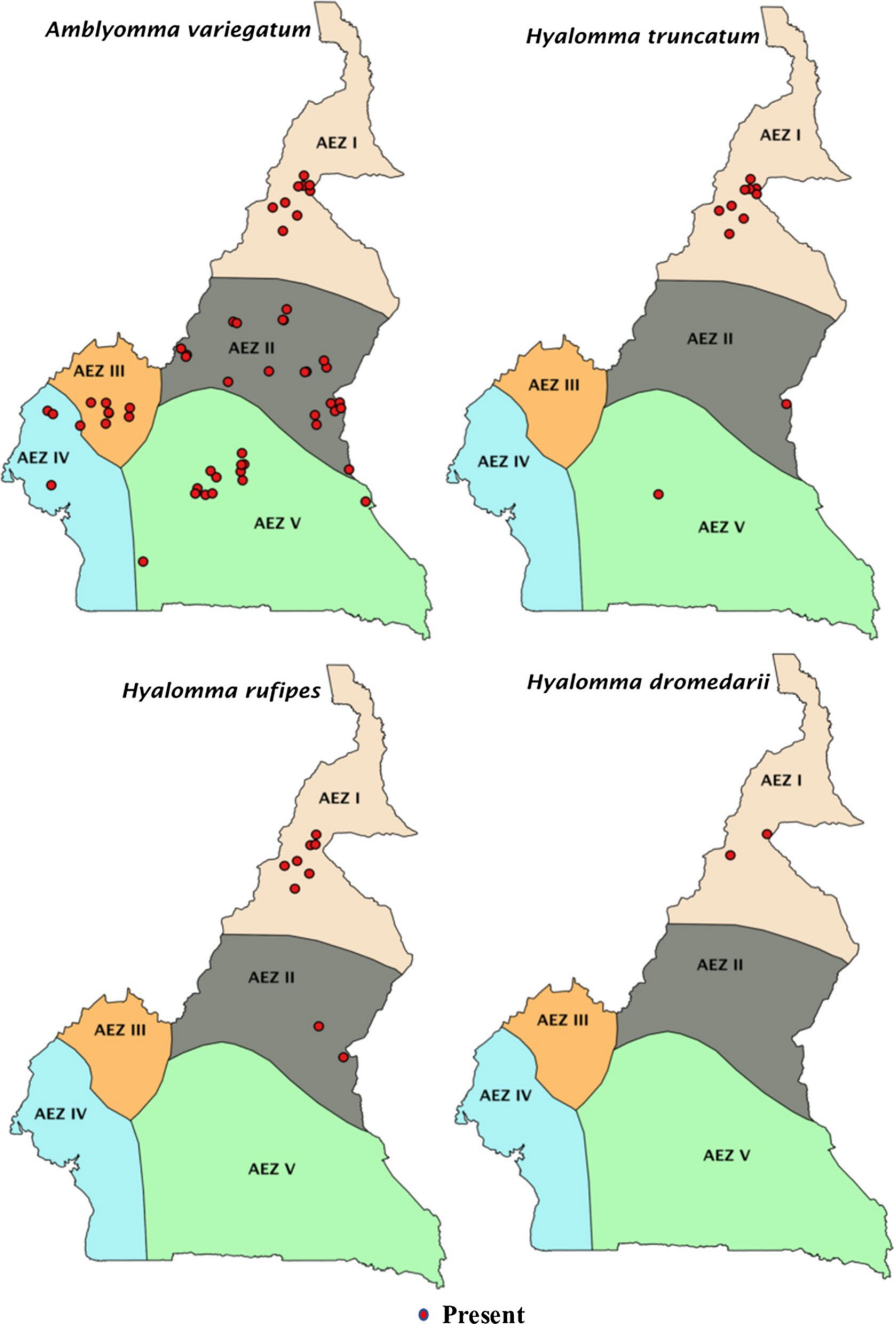
Geographical distribution of A. *variegatum*, *H. truncatum*, *H. rufipes* and *H. dromedarii*

**Fig. 4:**
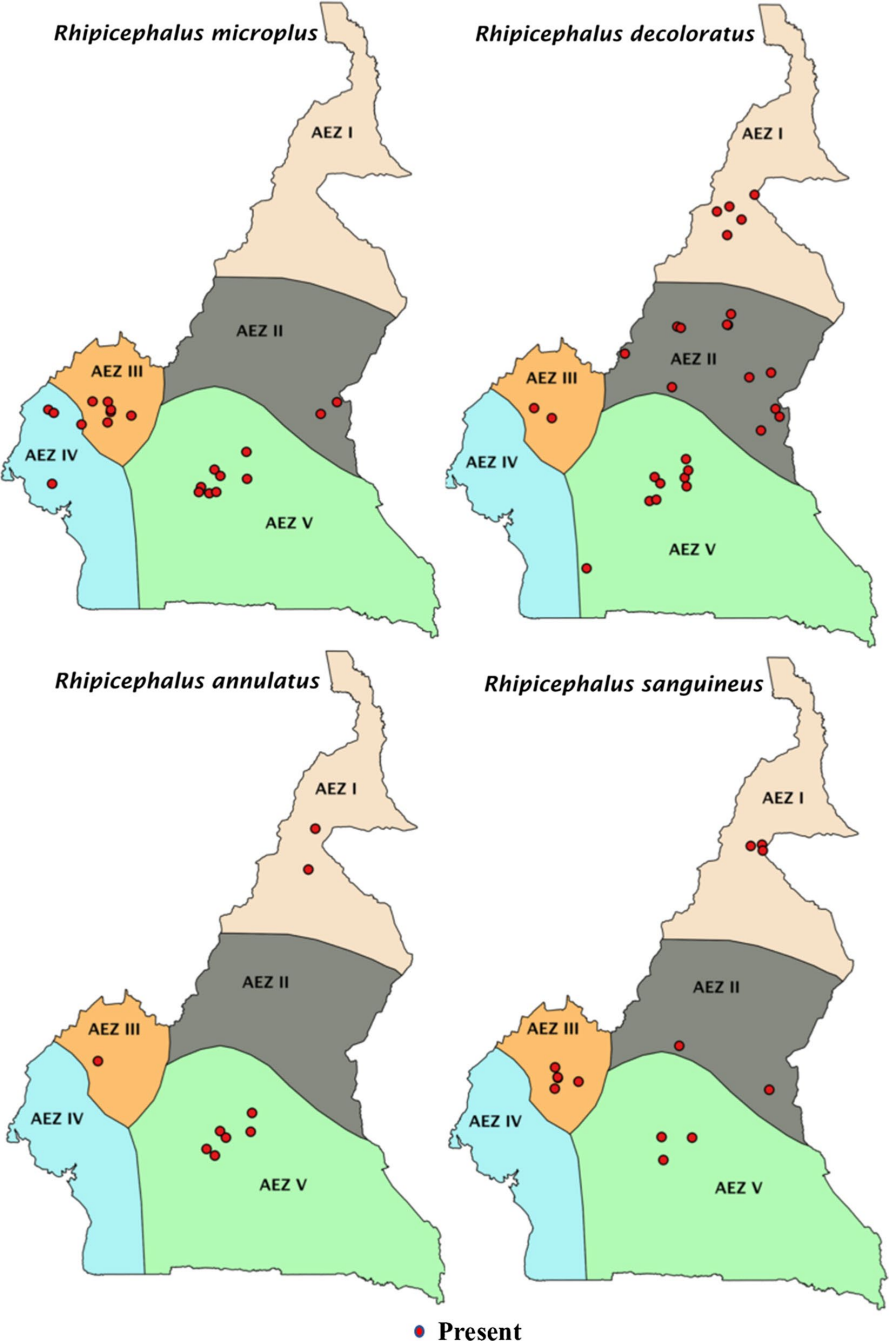
Geographical distribution of *R. microplus*, *R. decoloratus*, *R. annulatus* and *R. sanguineus*

*Amblyomma variegatum* outnumbered all the other tick species and represented 59.4% of the total tick collection (Table 2). This tick species was found at all sampling sites (Fig. 3). The overall abundance of *R. microplus* was 15.6%, making it the second most frequently observed tick species*. Rhipicephalus microplus* represented 51.1%, 65.2% and 38.3% of ticks collected in zones III, IV and V, respectively. These three zones represent the areas invaded by *R. microplus*, comprising a total of 25 sampling sites. When comparing the occurrence of *R. microplus* and *R. decoloratus* species in the areas invaded by *R. microplus* (zones III, IV and V), *R. microplus* was present at 18 sites while *R. decoloratus* was only present in 11 sites. Interestingly, *R. microplus* was recorded at all the sampling localities within the humid forest zone with monomodal rainfall (zone IV) where no *R. decoloratus* was found (Table 4). In the Western highlands, 8 localities were sampled and *R. decoloratus* was recorded in fewer localities (*n* = 2) than *R. microplus* (*n* = 7). Overall, *R. microplus* and *R. decoloratus* were sympatric at 8 of the 25 sites. *Rhipicephalus microplus* was found singly in 10 sites, whereas only 3 sites had *R. decoloratus* but not *R. microplus*. *Rhipicephalus annulatus* was collected mainly in AEZs V and AEZ I while *H. rufipes*, *H. dromedarii* and *H. truncatum* were observed in AEZ I and II where annual average rainfall is low (Figs. 3, 4).

**Table 4:** Occurrence data for *R. microplus* and *R. decoloratus* from Cameroon

AEZ	Nsite	Ncattle	Ntick	*R. microplus*		*R. decoloratus*		Sympatric
				*n* (%)	l (%)	*n* (%)	l (%)	l (%)
AEZ I	9	99	1667	0 (0)	0 (0)	167 (10.0)	5 (55.5)	0 (0)
AEZ II	20	238	2834	5 (0.2)	2 (10)	73 (2.6)	12 (60)	0 (0)
AEZ III	8	95	564	288 (51.1)	7 (87.5)	18 (3.2)	2 (25)	2 (25)
AEZ IV	3	20	158	103 (65.2)	3 (100)	0 (0)	0 (0)	0 (0)
AEZ V	14	149	1868	716 (38.3)	8 (57.1)	450 (24.1)	9 (64.2)	6 (42.8)
Total	54	601	7091	1112 (15.6)	20 (37)	708 (10.0)	28 (51.8)	8 (14.8)

*Abbreviations*: Nsite, total number of sites visited; Ncattle, total number of cattle sampled; Ntick, total number of ticks collected (all species); *n*, number of ticks collected; l, number of sites where the tick is present

### Phylogenetic analysis of the *cox*1 gene

**Fig. 5:**
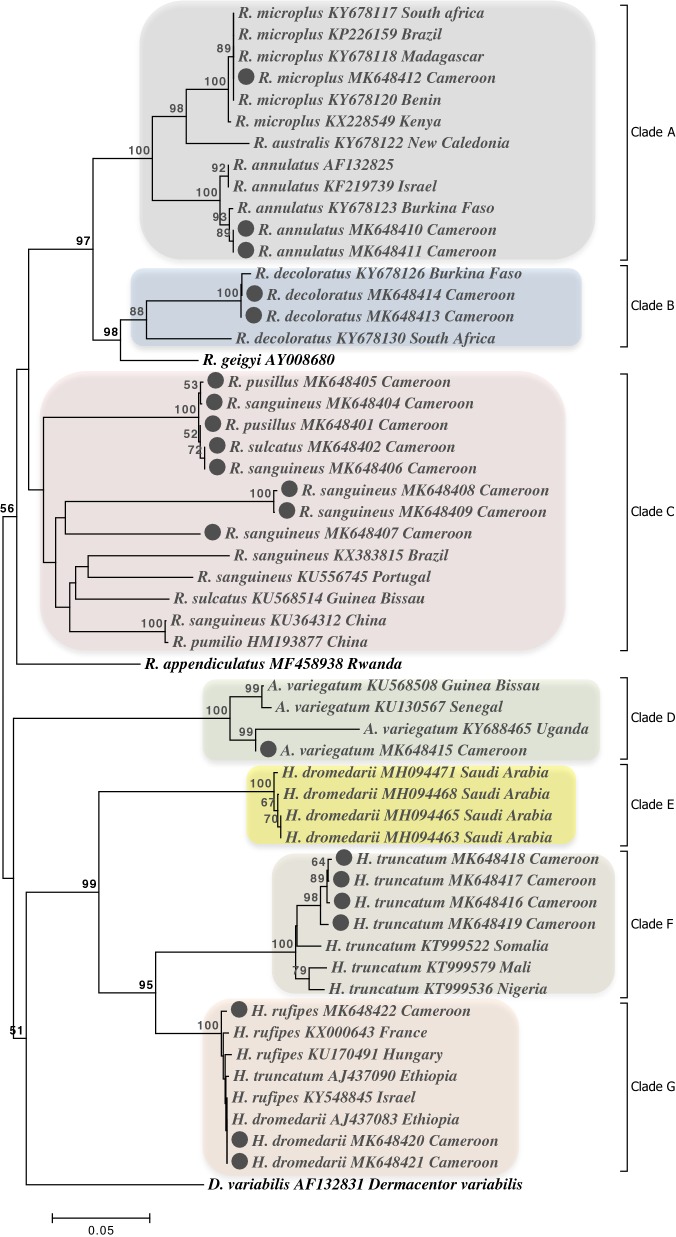
Phylogenic analysis of *cox*1 sequences of ticks from Cameroon. The evolutionary history was inferred by using the neighbor-joining method. The percentage of trees in which the associated taxa clustered together is shown above the branches. Major clades are indicated by the letters A, B, C, D, E, F and G. Sequences from this study are highlighted

Twenty-two *cox*1 sequences representing all the identified tick species generated in the present study and 34 reference *cox*1 sequences from various tick species present in the GenBank database were used to infer phylogenetic relationships between the tick taxa. The neighbor-joining tree resulting from mitochondrial *cox*1 sequences derived from the Hyalomminae, Rhipicephalinea and Amblyomminae sub-families consisted of seven clades (A, B, C, D, E, F, G) that have strong bootstrap support (98–100%) at the majority of nodes (Fig. 5). Clade A constitutes the ‘*R. microplus* complex’. Clades B and D each contain individuals within the same species, well supported by bootstrap values of 98 and 100, respectively. Clade C comprises members of the ‘*R. sanguineus* group’. Clade E comprises *H. dromedarii* from Saudi Arabia while clade F includes *H. truncatum* from Cameroon collected in the present study and also *H. truncatum* from Nigeria, Somalia and Mali. Clade G comprises more than one species, including *H. rufipes* and *H. dromedarii* from the present study, *H. rufipes* from France, Hungary and Israel, and *H. truncatum* and *H. dromedarii* from Ethiopia.

## Discussion

Few studies have focused on ticks infesting cattle in Cameroon and, as a result, there is limited data on the extent of tick burden and TBDs in the country. This is particularly concerning given the recent introduction and spread into West Africa of the highly invasive tick species *R. microplus* [12, 14, 15]. Moreover, cattle trade in the sub-region is largely unregulated [11], creating risks of disease dissemination that require detailed investigation [11]. Therefore, accurate data from field studies is urgently needed to inform rational control strategies and establish models to predict the changing epidemiology of TBDs, and the economic impact on livestock production.

From the present study, *A. variegatum*, the African bont tick and the main vector of *Ehrlichia ruminantium*, was ubiquitous in all sampling localities spanning an extremely divergent range of climatic conditions. This finding is consistent with previous studies that reported occurrence of *A. variegatum* across the entire country throughout the year [20, 25]. A similar situation has been described for small ruminants [18]. *Amblyomma variegatum* also transmits the protozoans *Theileria mutans* and *T. velifera* causing benign bovine theileriosis [42, 43]. This tick has also been associated with dermatophilosis caused by the bacteria, *Dermatophilus congolensis*. The disease can affect tick-free cattle but is more severe in cattle infested by *A. variegatum* [44]. The role of *A. variegatum* in the development of dermatophilosis was demonstrated to be promoted through immunosuppression that occurs after tick-feeding and predispose entry of the bacteria into the skin [45]. Given the high prevalence and wide distribution of *A. variegatum*, a future survey of the impact of ehrlichiosis in the livestock sector in Cameroon would be well justified.

An immediate effect of the lack of surveillance at national borders has been the introduction of *R. microplus* in southern Cameroon, seemingly from Nigeria [46]. Of serious concern, *R. microplu*s was found to be the second most abundant species. This finding contrasts with recent reports from North Central Nigeria and Cameroon which concluded that *R. microplus* was absent and *R. decoloratus* was the most abundant tick species in the areas studied [19, 47]. It is worth noting that the exotic *R. microplus* already outnumbers the indigenous *R. decoloratus* in our study, attesting to the aggressive nature of this species. Interestingly, in the areas invaded by *R. microplus* which were within AEZs III, IV and V, *R. microplus* was present in more sites than *R. decoloratus* (Table 4). This rapid expansion of *R. microplus* followed by apparent displacement of *R. decoloratus* in the field is likely attributable to the shorter life-cycle and higher egg production capacity of *R. microplus* [48]. This phenomenon has recently been reported in several African countries, including Tanzania and South Africa, both likely originating from independent introductions, and also Ivory Coast [49–51]. Moreover, the ability of *R. microplus* to develop resistance to most available acaricides might also have favored its expansion at the expense of more susceptible species [52]. In this scenario, the displacement of *R. decoloratus* by *R. microplus* could rather be due to the pressure exerted by acaricides on the former species. A similar situation has been described in the leafminer fly [53]. It is also the case that these two hypotheses are not mutually exclusive, and both factors may have contributed to the displacement of *R. decoloratus* by *R. microplus*. Regardless of the explanation for the changing dynamics of these two tick populations, the apparent displacement between the two species highlights the need to accurately identify species circulating in the field and adjust the management strategies accordingly.

The observed sex ratio of the tick species collected in the present study varied from one species to another. The male to female sex ratio in *A. variegatum*, *H. truncatum* and *H. rufipes* showed that males were present in greater number than females. These results are in agreement with previous reports [54–56]. This suggest that males remain on the host for a longer period than females which drop off to the ground to lay eggs when they are fully engorged [57]. The low male to female ratio observed for *R. microplus* and *R. decoloratus* could imply that males were not collected due to their small size, since only visible adults were picked during the sampling.

In general, the mean tick burden in the country was low. This may not reflect the reality in the field since the collection included only adult ticks. This points to a need for a quantitative longitudinal survey with complete collection that include all tick stages. The lowest tick burden in highlands (AEZIII) compared to the northern plains and the Adamawa plateau agreed with previous findings and may be attributed to the mortality of a high proportion of tick instars due to low temperatures in highlands [58].

Recently, tracking the spatial movement of transhumant herds in Cameroon, Motta et al. [9] demonstrated that seasonal migration of most of the herds originating from AEZ II occurred in the direction of the areas invaded by *R. microplus* (AEZ V). Therefore, it is worth noting that transhumance exposes herds to *R. microplus* infestation and consequently to bovine babesiosis, a serious threat to the livestock production.

*Hyalomma* species were commonly encountered in dry habitats (AEZ I and II), in line with the known distribution of these species [39]. The presence of a single individual of *H. truncatum* in AEZ V could be the result of unusual cattle migration, or bird migration as previously suggested [23, 39]. Previous studies did not report *H. rufipes* in Northern Cameroon [18]. The present survey reveals a relatively high abundance (12.4%) of *H. rufipes* in AEZ I (Table 2), which is of great public health importance since *H. rufipes* is one of the main competent vector of the virus causing Crimean-Congo hemorrhagic fever (CCHF) in humans [59]. This tick species also transmits *Anaplasma marginale* and *Babesia occultans* to cattle and *Rickettsia conorii* to humans, the latter of which being the causal agent of Mediterranean spotted fever [39, 60].

*Hyalomma dromedarii* was reported for the first time in Cameroon. This finding is of great veterinary and public heath importance, since the tick is an additional vector of CCHF virus and can also transmit *Theileria annulata* to cattle [39, 61, 62]. To the best of our knowledge, *H. dromedarii* has never previously been reported in Cameroon. Nevertheless, it appears in a list of ticks considered likely to occur in Cameroon published in 1958 [25].

The phylogenetic analysis of *cox*1 sequences from *H. truncatum*, *H. dromedarii* and *H. rufipes* revealed that none of these *Hyalomma* species form a monophyletic group according to analysis of *cox*1 gene sequences. This observation could be explained by introgressive hybridization where one ‘species’ incorporates genes into the gene pool of another ‘species’ as a consequence of ‘interspecific hybridization’. This lack of concordance between morphology and molecular identification has been demonstrated previously and has been attributed to hybridization between African *Hyalomma* taxa [63]. Such data raise fundamental issues as to what constitutes a true species and may require application of additional techniques, such as sequencing of specific genes encoded in the nuclear genome, genome-wide SNP analysis using next generation sequencing and mass spectrometry [64].

Due to the high incidence of morphological similarity and existence of cryptic species, the members of the subfamily Rhipicephalinae are often clustered within species complexes [65]. In the present study, phylogenetic analysis within this group revealed that clade C very likely contains more than one species (Fig. 5). The existence of different species classified under the generic term ‘*R*. *sanguineus* group’ has recently been reported [66]. This suggests that the members of Rhipicephalinae are often misidentified. Therefore, there is a need of further genetic and phenotypic analyses as outlined above in order to clarify the taxonomic status and genetic relationships among members of these complexes.

Comparing our results with previous studies on ticks infesting cattle in Cameroon, *R. microplus* and *H. dromedarii* have been added to the known list of tick species present in the country. This is of both veterinary and public health importance since these species are major ixodid vectors of human and livestock diseases worldwide [61, 67]. Furthermore, *R. microplus* is known to rapidly develop resistance to most classes of acaricides [68]. It seems that recent reports relating to increased tick infestation and acaricide application in some areas of Cameroon are the result of *R. microplus* resistance to locally used acaricides (manuscript in preparation). To fill this knowledge gap, further research on tick resistance or susceptibility to locally used acaricides needs to be undertaken to inform and advise farmers and stakeholders on sustainable control methods, which may differ depending on the presence of *R. microplus*.

This cross-sectional survey reveals the tick species circulating in Central and Eastern Cameroon (AEZ V), an understudied area as far as TBDs are concerned. Nonetheless, it is possible that the full extent of the geographical ranges of these tick species have not yet been defined. This cross-sectional survey cannot differentiate temporary tick populations from well-established populations. *Rhipicephalus microplus* was the most abundant tick (38.3%) in AEZ V which borders C.A.R. It is important to note that in a recent review of ticks in C.A.R., *R. microplus* was not reported at all [69]. These findings show the potential risk of *R. microplus* being introduced into C.A.R., since farmers who migrated to Cameroon with their livestock during the recent period of political instability may return to their country, resulting in further transboundary spreading of the tick.

## Conclusions

The present study provides the first comprehensive overview of the current distribution of cattle ticks in Cameroon. Mapping tick occurrence provides a solid basis for identifying areas where herds are at risk of being exposed to *R. microplus* infestation and related TBDs. The information presented herein could help to define transhumance corridors which might potentially limit herd exposure to *R. microplus*. The data generated will also be useful in informing a targeted tick control strategy in a more sustainable, environmentally compatible and cost-effective manner. Further longitudinal studies are envisaged to determine whether the tick species identified in the current horizontal survey are established or transient and what factors contribute to the ongoing displacement of the native African tick species by the invasive *R. microplus.*

## Supplementary information

**Additional file 1: Table S1.** Geographical coordinates, BLASTn results and GenBank accession numbers of cytochrome oxidase I (*cox*1) gene sequences of cattle ticks from Cameroon.

**Additional file 2: Figure S1.** *Amblyomma variegatum* and *Rhipicephalus sanguineus* dorsal and ventral views.

**Additional file 3: Figure S2.** *Hyalomma truncatum* and *Hyalomma rufipes* dorsal and ventral views.

**Additional file 4: Figure S3.** *Rhipicephalus annulatus* dorsal and ventral views.

**Additional file 5: Figure S4.** *Rhipicephalus annulatus*, adult female, hypostomal teeth.

**Additional file 6: Figure S5.** *Rhipicephalus annulatus*, adult female, palp articles.

**Additional file 7: Figure S6.** *Rhipicephalus decoloratus* dorsal and ventral views.

**Additional file 8: Figure S7.** *Rhipicephalus decoloratus*, adult female, hypostomal teeth.

**Additional file 9: Figure S8.** *Rhipicephalus decoloratus*, adult female, palp articles.

**Additional file 10: Figure S9.** *Rhipicephalus microplus* dorsal and ventral views.

**Additional file 11: Figure S10.** *Rhipicephalus microplus*, adult female, hypostomal teeth.

**Additional file 12: Figure S11.** *Rhipicephalus microplus*, adult female, palp articles.

## Data Availability

The datasets used and/or analyzed during the present study are available from NCBI GenBank (accession numbers MK648401-MK648422).

## Abbreviations

*cox*1: cytochrome *c* oxidase subunit 1
TBDs: tick-borne diseases
C.A.R: Central African Republic
ITS: internal transcribed spacer
AEZs: agro-ecological zones
