# Supplementary material for: A comprehensive survey of the prevalence and spatial distribution of ticks infesting cattle in different agro-ecological zones of Cameroon
Source: Parasit Vectors. 2019 Oct 17;12:489. doi: 10.1186/s13071-019-3738-7 (PMC6796472; doi:10.1186/s13071-019-3738-7)
Supplement: Supplementary file 2 — Additional file 2: Figure S1. Amblyomma variegatum and Rhipicephalus sanguineus dorsal and ventral views. [file 13071_2019_3738_MOESM2_ESM.pdf]

Amblyomma variegatum

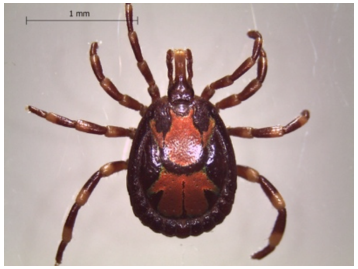

Adult male, dorsal

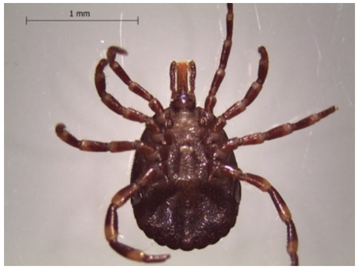

Adult male, ventral

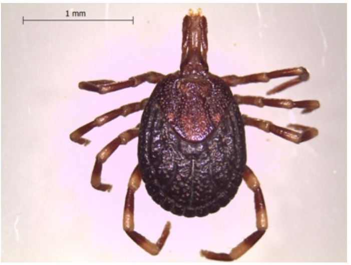

Adult female, dorsal

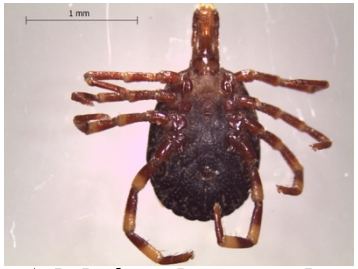

Adult female, ventral

Rhipicephalus sanguineus

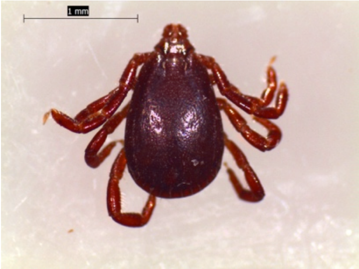

Adult male, dorsal

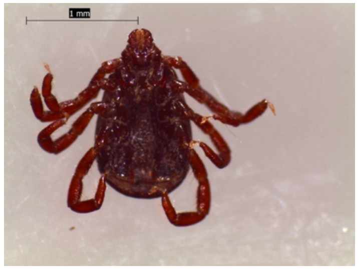

Adult male, ventral

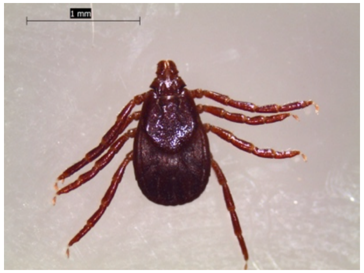

Adult female, dorsal

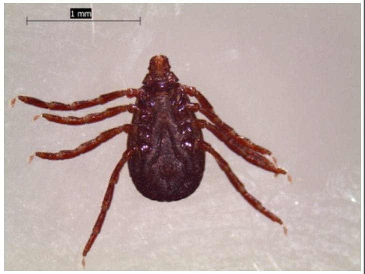

Adult female, ventral
