# Supplementary material for: A comprehensive survey of the prevalence and spatial distribution of ticks infesting cattle in different agro-ecological zones of Cameroon
Source: Parasit Vectors. 2019 Oct 17;12:489. doi: 10.1186/s13071-019-3738-7 (PMC6796472; doi:10.1186/s13071-019-3738-7)
Supplement: Supplementary file 3 — Additional file 3: Figure S2. Hyalomma truncatum and Hyalomma rufipes dorsal and ventral views. [file 13071_2019_3738_MOESM3_ESM.pdf]

Hyalomma truncatum

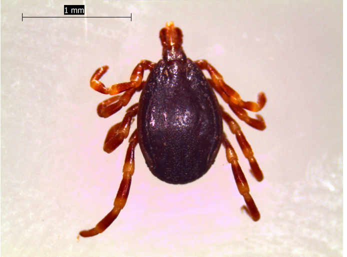

Adult male, dorsal

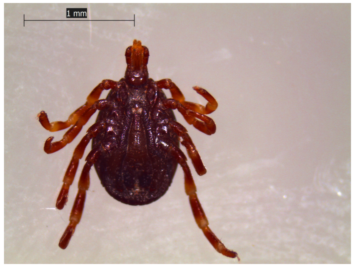

Adult male, ventral

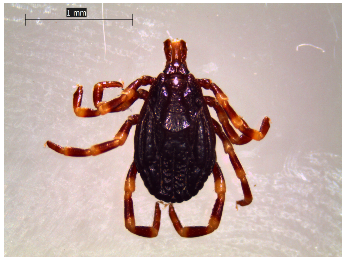

Adult female, dorsal

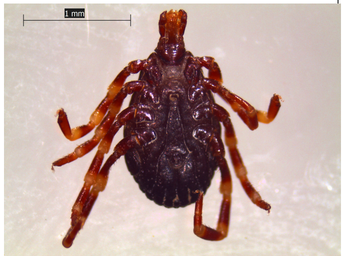

Adult female, ventral

Hyalomma rufipes

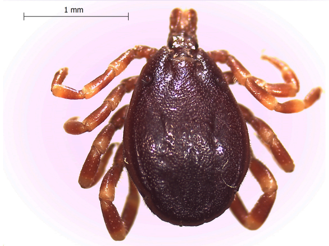

Adult male, dorsal

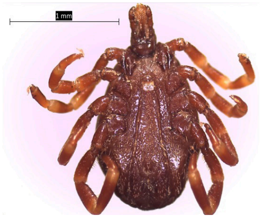

Adult male, ventral

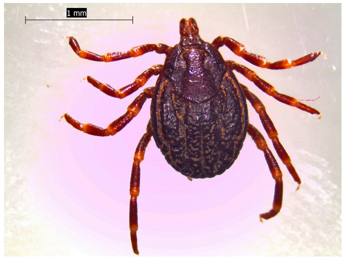

Adult female, dorsal

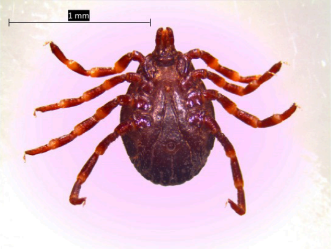

Adult female, ventral
