# Supplementary figures and images for: A comprehensive survey of the prevalence and spatial distribution of ticks infesting cattle in different agro-ecological zones of Cameroon
Source: Parasit Vectors. 2019 Oct 17;12:489. doi: 10.1186/s13071-019-3738-7 (PMC6796472; doi:10.1186/s13071-019-3738-7)

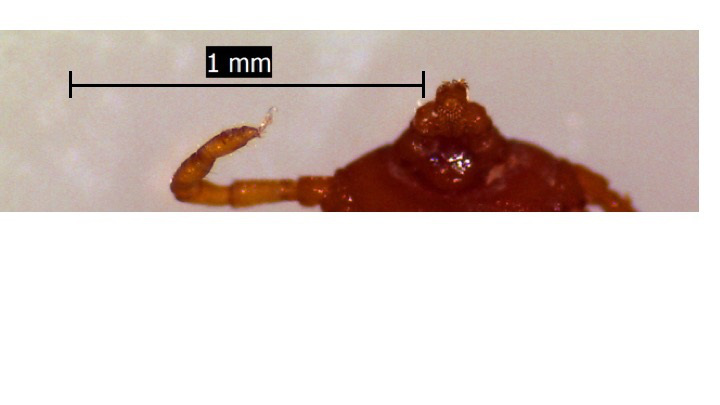

Supplement: Supplementary file 5 — Additional file 5: Figure S4. Rhipicephalus annulatus, adult female, hypostomal teeth. [file 13071_2019_3738_MOESM5_ESM.jpeg]

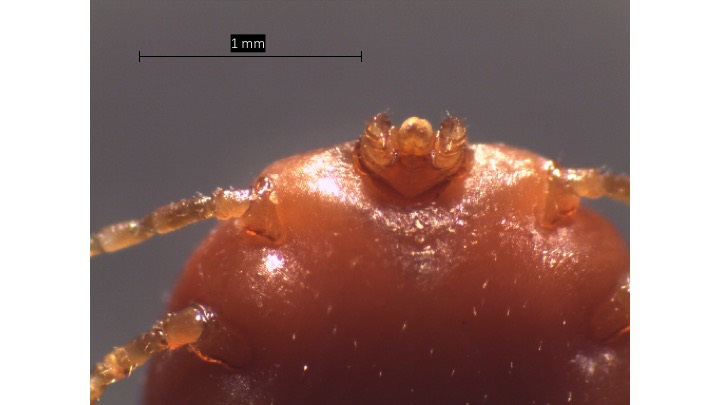

Supplement: Supplementary file 6 — Additional file 6: Figure S5. Rhipicephalus annulatus, adult female, palp articles. [file 13071_2019_3738_MOESM6_ESM.jpeg]

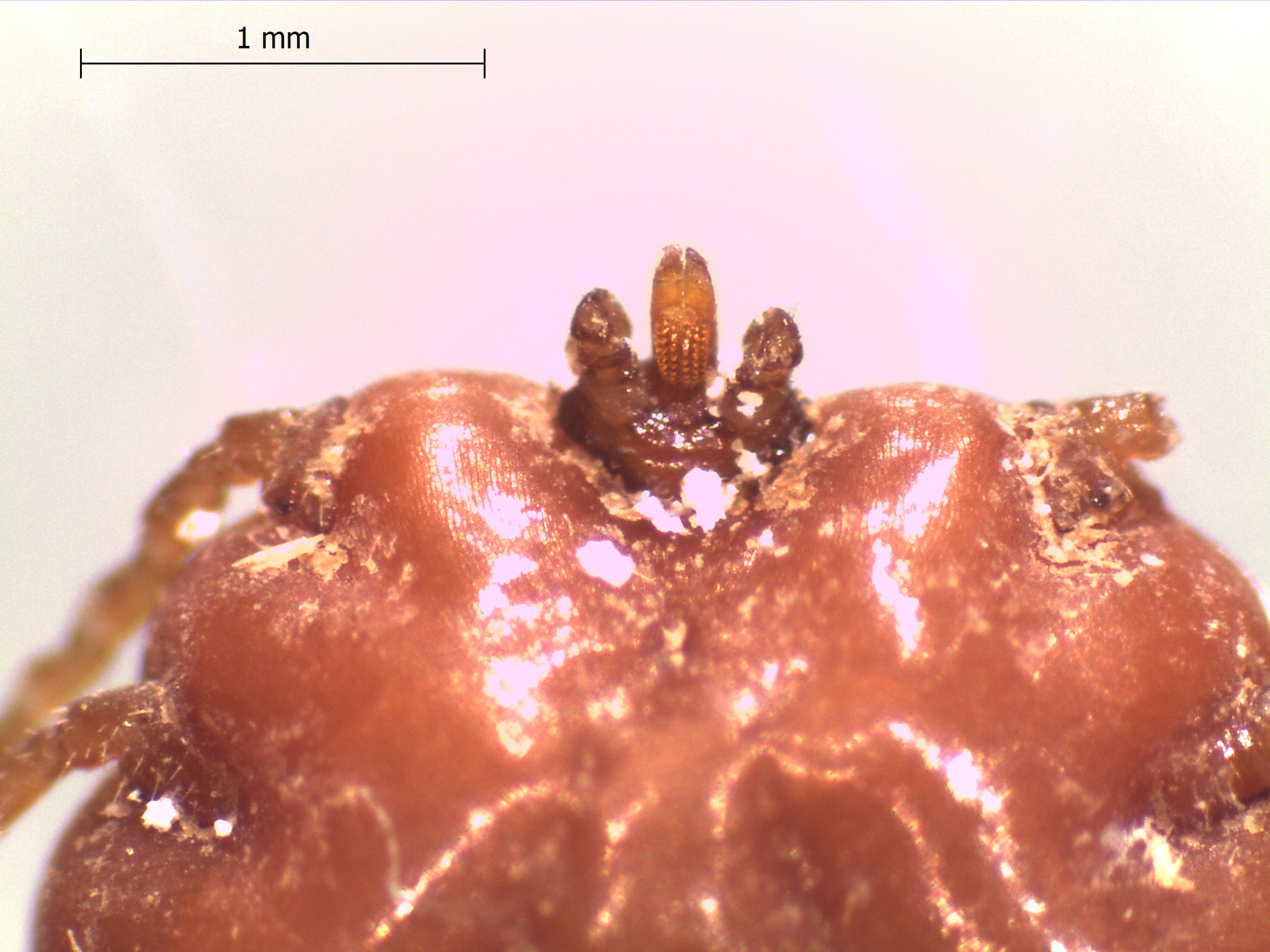

Supplement: Supplementary file 8 — Additional file 8: Figure S7. Rhipicephalus decoloratus, adult female, hypostomal teeth. [file 13071_2019_3738_MOESM8_ESM.jpg]

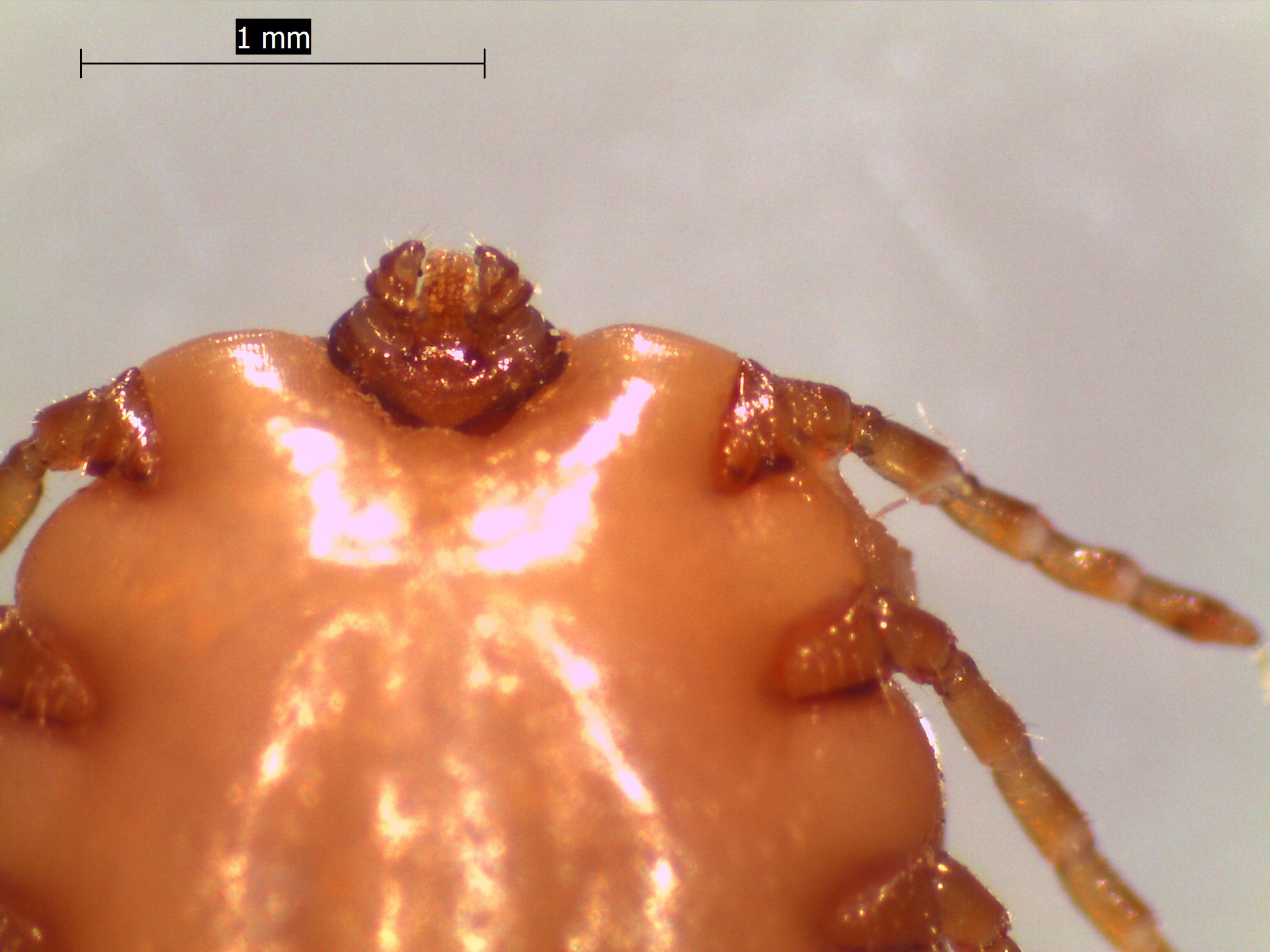

Supplement: Supplementary file 9 — Additional file 9: Figure S8. Rhipicephalus decoloratus, adult female, palp articles. [file 13071_2019_3738_MOESM9_ESM.jpg]

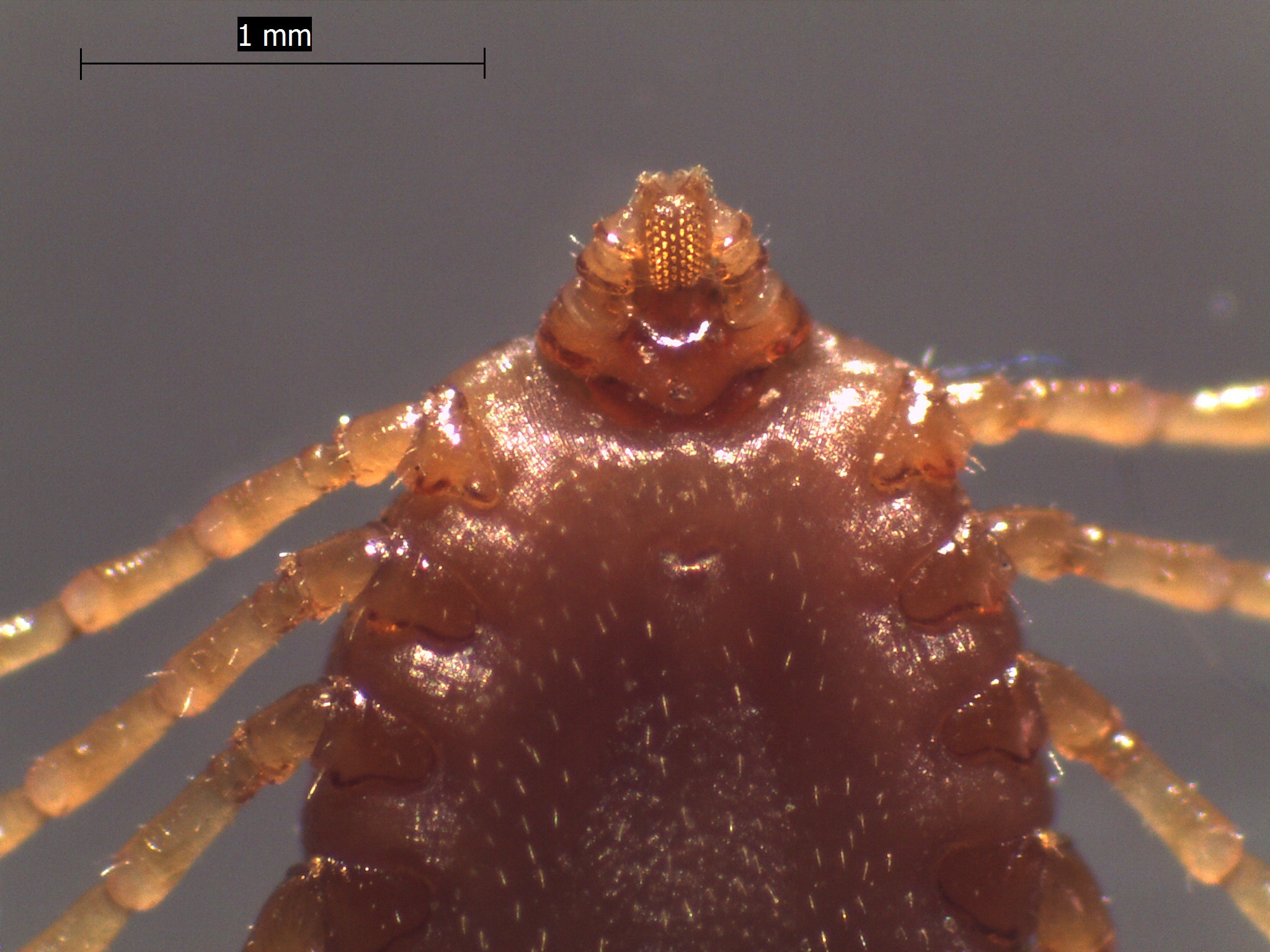

Supplement: Supplementary file 11 — Additional file 11: Figure S10. Rhipicephalus microplus, adult female, hypostomal teeth. [file 13071_2019_3738_MOESM11_ESM.jpg]

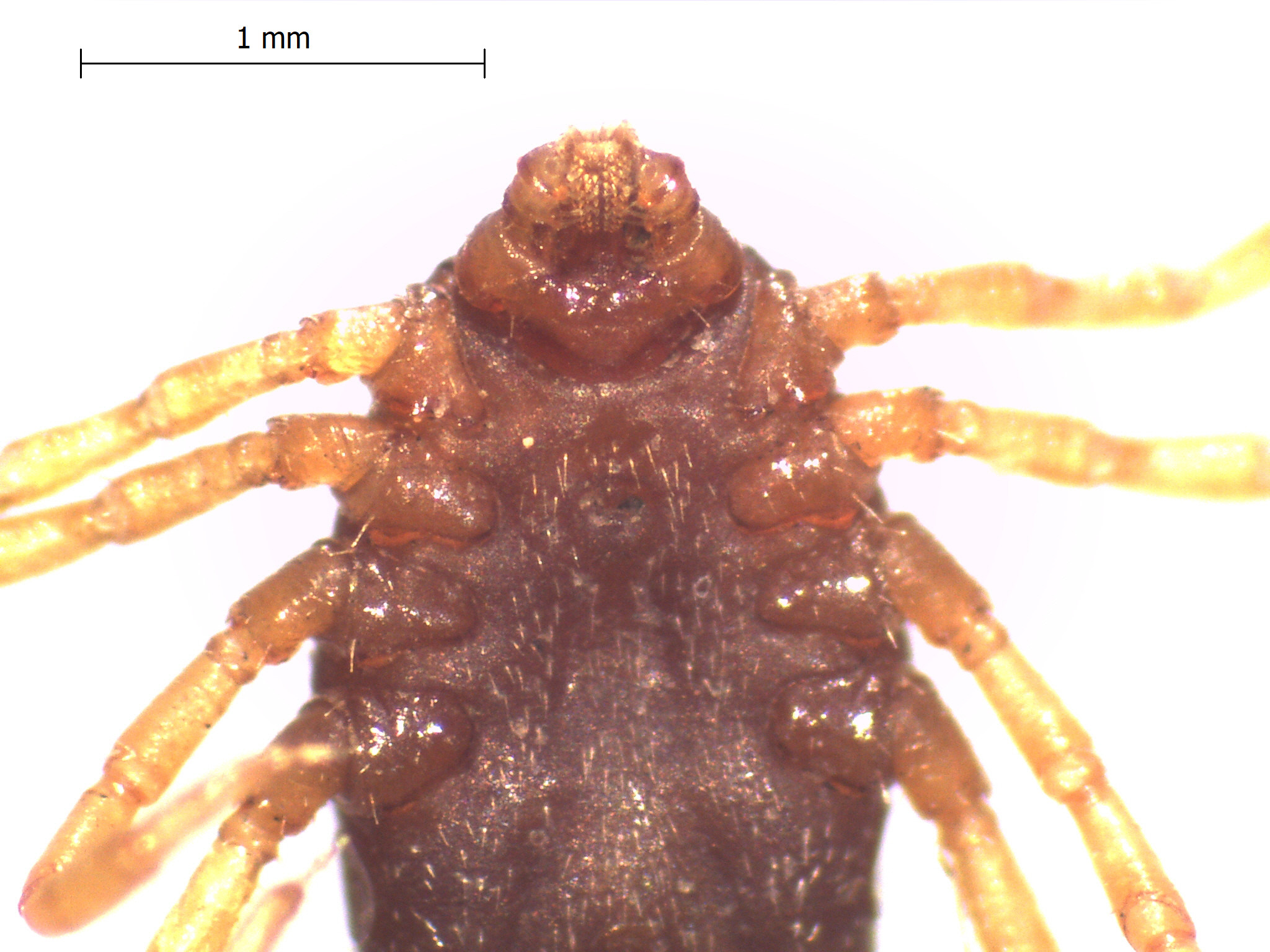

Supplement: Supplementary file 12 — Additional file 12: Figure S11. Rhipicephalus microplus, adult female, palp articles. [file 13071_2019_3738_MOESM12_ESM.jpg]
