# Supplementary material for: A comprehensive survey of the prevalence and spatial distribution of ticks infesting cattle in different agro-ecological zones of Cameroon
Source: Parasit Vectors. 2019 Oct 17;12:489. doi: 10.1186/s13071-019-3738-7 (PMC6796472; doi:10.1186/s13071-019-3738-7)
Supplement: Supplementary file 10 — Additional file 10: Figure S9. Rhipicephalus microplus dorsal and ventral views. [file 13071_2019_3738_MOESM10_ESM.pdf]

Rhipicephalus microplus

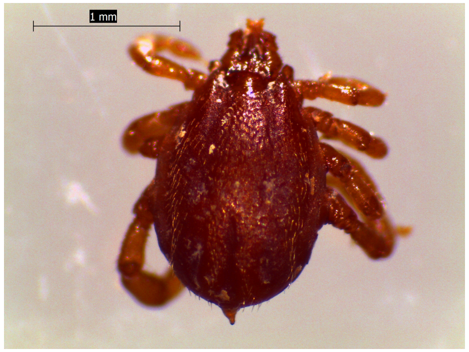

Adult male, dorsal

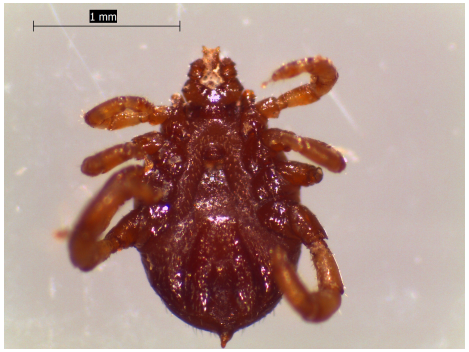

Adult male, ventral

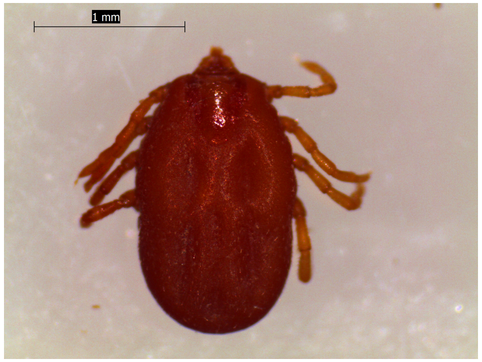

Adult female, dorsal

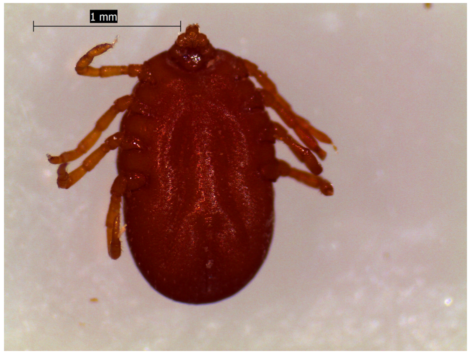

Adult female, ventral

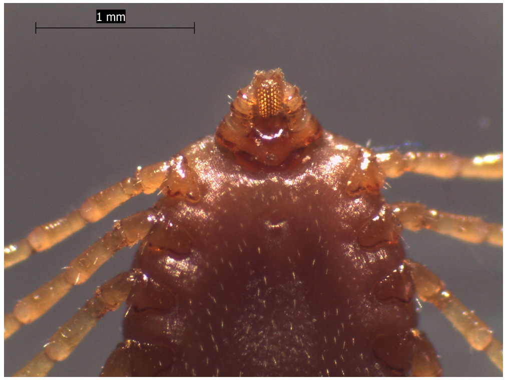

Adult female, genital aperture

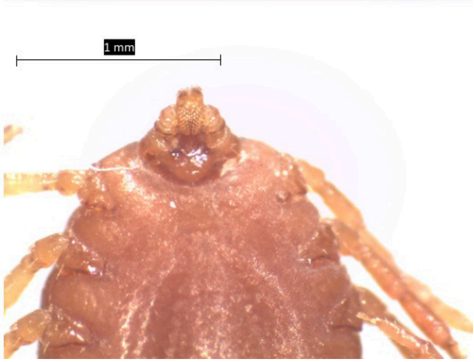

Adult female, coxae 2 and 3 spurs are present
